# Genomic Characterisation of *Campylobacter jejuni* Isolates Recovered During Commercial Broiler Production

**DOI:** 10.3389/fmicb.2021.716182

**Published:** 2021-10-14

**Authors:** Brendha Truccollo, Paul Whyte, Catherine M. Burgess, Declan J. Bolton

**Affiliations:** ^1^Food Safety Department, Teagasc Food Research Centre, Dublin, Ireland; ^2^School of Veterinary Medicine, University College Dublin, Dublin, Ireland

**Keywords:** *Campylobacter*, broilers, thinning, genomic characterisation, MLST, virulence genes, antimicrobial resistance

## Abstract

**Background:** *Campylobacter* is commonly transmitted to humans from chickens. *Campylobacter jejuni* is the species most frequently associated with human illness, and the most prevalent species recovered from poultry.

**Objective:** The objective of this study was to analyse a sub-population of *C. jejuni* from two broiler flocks on the farm and at slaughter using whole-genome sequencing to gain insights into the changes in the *Campylobacter* population during broiler production, including changes in virulence and antimicrobial resistance profiles.

**Methods:** In this study, ten composite faecal samples (*n*=10), obtained by pooling ten fresh faecal samples (*n*=10), were collected in the broiler house on two farms on days 14, 21, 28, and 34 (*n*=80) and ten composite (*n*=10) caecal samples were collected at the time of slaughter for each flock (*n*=20). These were tested for *C. jejuni* using the ISO 10272-2:2016 method. Seven isolates were randomly selected from each of the nine *Campylobacter*-positive sampling points (*n*=63) and were subjected to antimicrobial susceptibility tests. Their genomes were sequenced and the data obtained was used to characterise the population structure, virulence, antimicrobial resistance determinants and inter-strain variation.

**Results:** The Farm 1 isolates had three MLST types (ST257-257, ST814-661 and ST48-48) while those on Farm 2 were ST6209-464 and ST9401. Interestingly, only the MLST types positive for most of the virulence genes tested in this study persisted throughout the production cycle, and the detection of antimicrobial resistance determinants (*gyrA* T86I and *tetO*) increased after thinning and at slaughter, with the detection of new strains.

**Conclusion:** The persistence of the most virulent strains detected in this study throughout the production cycle has important implications for the risk to consumers and requires further investigation. The detection of new strains within the population corresponding with the time of thinning and transportation reflects previous reports and provides further evidence that these activities pose a risk of introducing new *Campylobacter* strains to broiler batches.

## Introduction

*Campylobacter* is the most common cause of bacterial foodborne illness and it is most frequently transmitted to humans from poultry, causing over 200,000 reported cases in the European Union annually (EFSA, 2019). Most infections are self-limiting and symptoms of enteritis can range from mild and asymptomatic to severe, generally first appearing within 48h of ingestion and subsiding after 7 to 10 days without medical intervention (Blaser and Engberg, 2008; Kaakoush et al., 2015). More serious complications can emerge in rare cases, including the development of Guillain-Barré Syndrome, Miller Fisher Syndrome, reactive arthritis or bacteraemia (Kaakoush et al., 2015). *Campylobacter* is generally detected in broilers after 14 days of production, frequently reaching levels over 10^10^CFU/g in the broiler caecum and persists until slaughter, where consumer exposure can compromise public health (Callicott et al., 2006; Hermans et al., 2011).

Previous studies document the persistence, prevalence, virulence and antimicrobial resistance of *C. jejuni* in poultry and human isolates (Desmonts et al., 2004; Talukder et al., 2008; Frazão et al., 2017; EFSA, 2019); however, there are few reports that investigate these parameters in *C. jejuni* throughout the broiler production cycle. Increasing resistance to antimicrobials of clinical relevance, including macrolides and fluoroquinolones, has been reported in several countries (Liu et al., 2019; Ocejo et al., 2019). The acquisition of antimicrobial resistance determinants can occur as a result of spontaneous mutations or due to horizontal gene transfer, facilitated in environments that favour *C. jejuni* growth, such as broiler farms, particularly in the presence of selective pressure when antimicrobial administration is required (Davies and Wales, 2019). Additionally, the manifestation of campylobacteriosis in humans is dependent on the virulence of the infecting strain and on the health of the host. Although *Campylobacter* lacks classical virulence factors, such as endo- or enterotoxins, colonisation and survival determinants associated with motility, adhesion, invasion, capsule and biofilm synthesis, cytolethal distending toxin synthesis, molecular mimicry and secretion systems can result in the manifestation of more severe illness and persistence in the environment (Bolton, 2015; Stetsenko and Efimochkina, 2018; Wysok et al., 2020). Therefore, a more in-depth understanding of the changes in strains, virulence and antimicrobial resistance during broiler production would facilitate a more accurate assessment of the risk to public health associated with the broiler rearing cycle.

Previous studies have reported increasing antimicrobial resistance trends, and recently, the detection of more aerotolerant *C. jejuni* isolates in the latter stages of the production cycle has also been reported (Oh et al., 2015; Yao et al., 2016; Mouftah et al., 2021). Additionally, increased motility has been observed following *C. jejuni* passage through an animal host (Sang et al., 1989; Kim et al., 2012). These findings suggest that host interactions and persistence in key environments may contribute to the selection of strains with greater pathogenicity for humans.

The objective of this study was to perform a genomic characterisation of the *Campylobacter* population during broiler rearing, including the occurrence of virulence and antimicrobial resistance determinants.

## Materials and Methods

### Sample Collection

Two intensive broiler farms (*n*=2) were selected for this study, which was carried out between June and August 2018 (Farm 1) and between February and April 2020 (Farm 2). Both farms were located in county Monaghan in the northeast of Ireland. Each broiler house stocked approximately 30,000 broilers, *Gallus gallus domesticus* (Ross breed). Cattle farms were adjacent to both broiler houses. Faecal samples were collected on days 12, 21, 28 and 34 on both farms and caecal samples were collected in the abattoirs, after slaughter (day 38 for Farm 1 and day 40 for Farm 2). Thinning or partial depopulation occurred on day 30 on both farms. The faecal samples consisting of ten pooled samples containing ten fresh droppings each (*n*=100) were collected randomly using sterile scoops and sterile jars. The samples were transported to the laboratory in a cool, insulated carrier box and were processed within 4h of collection. On the day of slaughter, caecal samples consisting of ten pooled samples containing ten caeca each (*n*=100) were randomly collected from the slaughterhouse, transported in a cool box and processed within 24h.

### *Campylobacter* Spp. Enumeration

Enumeration was carried out in accordance with ISO 10272-2:2016. To 25g of each pooled faecal or caecal sample, 225ml of Bolton Broth (Oxoid, Basingstoke, United Kingdom) was added. Samples were stomached for 60s and serially diluted 10-fold in maximum recovery diluent (MRD, Oxoid, Basingstoke, United Kingdom). Each dilution was spread on modified charcoal cefoperazone deoxycholate agar (mCCDA, Oxoid, Basingstoke, United Kingdom) and incubated at 42°C microaerobically (10% CO_2_, 5% O_2_ and balancing N_2_) in a controlled atmosphere incubator (MACS VA-500, Don Whitley) for 48h.

### Confirmatory Tests

Five randomly selected presumptive colonies (*n*=5) were sub-cultured from each sample on Mueller Hinton agar (MHA, Oxoid, Basingstoke, United Kingdom) supplemented with 5% defibrinated horse blood and incubated at 42°C microaerobically for 48h. From each MHA culture, 2–3 colonies were inoculated into 2ml of Mueller Hinton broth (MHB; Oxoid, Basingstoke, United Kingdom) and incubated at 42°C microaerobically for 48h and DNA was subsequently extracted using the Qiagen DNeasy Blood and Tissue kit (Qiagen, Manchester, United Kingdom) following the manufacturer’s instructions. The species of each isolate was confirmed by PCR using a previously published method (Wang et al., 2002). The remaining growth on MHA was harvested into 1–2ml of defibrinated horse blood and stored at −80°C for future analyses.

### Antimicrobial Susceptibility

Antimicrobial susceptibility tests were carried out on 63 isolates in total (*n*=63), constituting seven randomly selected isolates (*n*=7) from each *Campylobacter* positive sampling point at each farm (*n*=49) and abattoir (*n*=14) using the broth microdilution method as per ISO 20776-1:2006 and EUCAST recommendations. Susceptibility to erythromycin (1–512mg/l), ertapenem (0.125–4mg/l), tetracycline (TET; 0.5–64mg/l), ciprofloxacin (CIP; 0.125–32mg/l), gentamicin (0.5–16mg/l) and chloramphenicol (2–64mg/l) was interpreted using EUCAST guidelines in accordance with ISO 20776-1:2006. Each isolate was grown in 10ml of MHB, which was incubated overnight at 42°C microaerobically. The inoculum was diluted 10-fold in 10ml of MH-F broth (clarified cation adjusted MHB with 20mg/l β-NAD and 5% lysed horse blood) to yield a solution with 1×10^6^CFU/ml. Fifty microlitres of this solution was added to each well containing 50μl broth containing double the desired antimicrobial concentration to yield a final inoculum of 5×10^5^CFU/ml. *C. jejuni* ATCC 33560 and *Staphylococcus aureus* ATCC 22391 were used as controls as per EUCAST recommendations. *C. jejuni* plates were incubated at 42°C microaerobically for 24–48h. *S. aureus* was incubated at 37°C aerobically for 24h. Resistance and susceptibility were determined using epidemiological cut-off points in accordance with EFSA recommendations (Aerts et al., 2019).

### Whole-Genome Sequencing

The same isolates as above (*n*=63) from Farms 1 and 2 were selected for genomic characterisation. Single colonies from mCCDA were sub-cultured into MHA and incubated at 42°C microaerobically for 48h. A loopful from each culture was suspended in PBS, gently mixed and centrifuged at 5000×*g* for 5min. The supernatant was discarded and 180μl of buffer ATL and 20μl of Proteinase K were added to each pellet. The remaining steps of the DNA extraction were followed per the manufacturer’s instructions (DNA Blood and Tissue Kit, Qiagen, Manchester, United Kingdom). RNA contamination was removed by incubating the samples at 37°C for 30min with 10μl of 100mg/ml RNase A (Thermo Fisher, Paisley, United Kingdom) after cell lysis, then returning the samples to 57°C for 30min. Each resulting DNA sample was quantified using the BR dsDNA kit with the Qubit 4.0 fluorometer (Thermo Fisher, Paisley, United Kingdom). Further quantification and 260:280 and 260:230 ratios were measured using the NanoDrop 2.0. Lastly, each sample was run on 1.0% agarose gel in 1X TBE buffer for 40min at 100V to check for DNA degradation. Samples with more than 500ng of DNA, free from RNA contamination and with a 260:280 ratio between 1.8 and 2 were subject to paired-end 150bp whole-genome sequencing on the Illumina NovaSeq 6,000 platform (Illumina, Cambridge, United Kingdom) with 100x coverage (Novogene, Cambridge, United Kingdom). Library preparation was performed by Novogene, Cambridge, United Kingdom, as per standard protocol using the NEBNext DNA Library Prep kit (New England Biolabs, Massachusetts, United States).

### Bioinformatic Analysis

The trimmed reads provided were assembled on SPades v. 3.14 (Bankevich et al., 2012) with the – careful parameter. Assembly quality was checked using QUAST (Gurevich et al., 2013) using *C. jejuni* NCTC 11168 as a reference. They were annotated on Prokka v. 2.14 (Seemann, 2014) with – use genus and – genus *Campylobacter* parameters.

The gff outputs from annotation were analysed in Roary (Page et al., 2015). The core genome alignment file output from Roary was used to construct a maximum likelihood tree using RAXML with m GTRGAMMA with 500 bootstraps (Stamatakis, 2014). Roary was also used to compare differences in the genes present in each strain using the query pan_genome command with – a difference, and differences in the genes present within each strain between sampling time points using the same command. The nucleotide sequences from genes and coding sequences (CDS) found to be uniquely present in a strain were extracted from pan_genome_reference.fa using query_pan_genome – a gene_multifasta. The outputs from strain vs. strain analyses were concatenated and submitted to eggNOG mapper v. 5.0 for functional annotation with a taxonomic scope restricted to Epsilonproteobacteria and standard parameters (Huerta-Cepas et al., 2017).

Multilocus sequence typing (MLST), which targets seven *C. jejuni* housekeeping genes, was carried out for each isolate using MLST^1^ equipped with the PubMLST database (Jolley et al., 2018). *cgMLST*, which targets of 1,300 core genes in *C. jejuni*, was also carried out to further distinguish the genomes analysed in this study. cgMLST alleles were first determined using cgMLST and cgSTs were determined using cgMLST^2^ Finder v. 1.1 (Clausen et al., 2018).

Abricate equipped with VFDB and Victors was used to extract all virulence genes from each isolate (Chen et al., 2016; Sayers et al., 2019). It was then equipped with Resfinder v. 4.1, CARD, ARG-ANNOT and NCBI AMR Finder to identify antimicrobial resistance determinants in each isolate (Gupta et al., 2014; Jia et al., 2017; Feldgarden et al., 2019). Hits with less than 80% identity and/or coverage were filtered out of the analysis.^3^

Assemblies were filtered to remove contigs <200bp using BBMap v. 38.22 (Bushnell, 2014) prior to submitting each genome into BioProject ID PRJNA688841.

### Statistical Analysis

Significant differences in the proportions of COG (Clusters of Orthologous Groups) categories between strains were calculated using a chi-square test in R Studio (Boston, MA, United States) equipped with R v. 3.6.3.

## Results

### Prevalence

**Figure 1:**
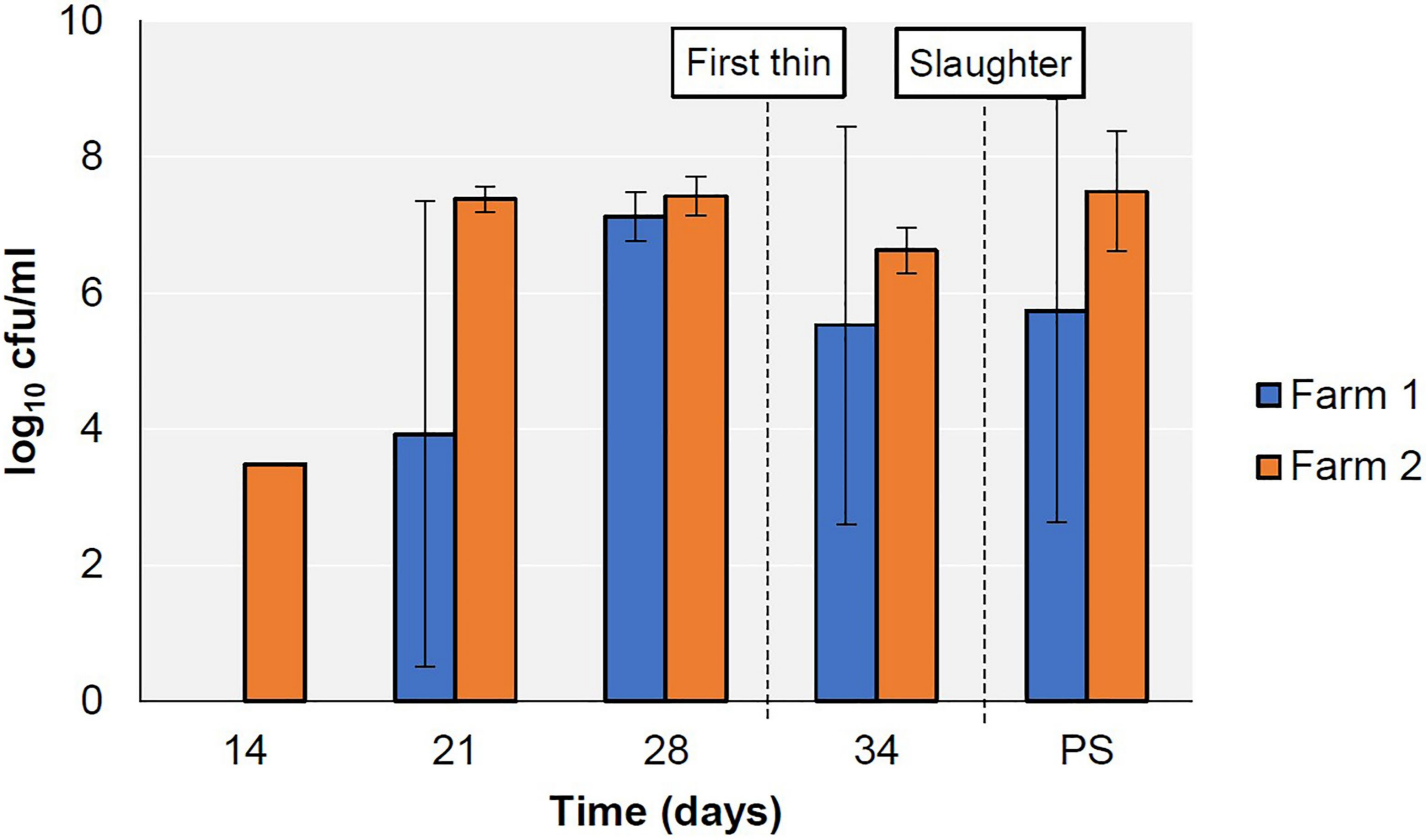
Prevalence of *Campylobacter* spp. in faecal (days 14–34) and caecal (PS; post-slaughter) samples from two broiler farms.

*Campylobacter* was first detected on Farm 1 on day 21 and on Farm 2 on day 14, persisted on both farms until the end of the production cycle (Figure 1), and was subsequently detected in the abattoir caecal samples. All *Campylobacter* isolates detected in this study were confirmed as *C. jejuni*.

### Genome Analysis

A total of sixty-three genomes were sequenced (*n*=63): twenty-eight from Farm 1 from days 21, 28 and 34 during the production cycle and the abattoir caecal samples (*n*=28) and thirty-five from Farm 2 from days 14, 21, 28 and 34 of the production cycle and the abattoir samples (*n*=35). Genomes were assembled with 27 contigs on average, ranging from 14 to 60. Genome lengths ranged from 1.6 to 1.8 Mbp, GC content was on average 30.3%, ranging from 30.07 to 30.47%. The genomes were assigned to five MLST types and six cgST types, and their mean number of base pairs is provided in Table 1.

**Table 1:** Average genome length per MLST and cgST type.

Source	MLST Type	MLST Type	Average length (bp; mean±SD)
Farm 1	*ST48-48 (*n*=14)*	*34286*	*1,641,757.25±579.77*
		*34272*	*1,641,350.50±951.06*
	*ST257-257 (*n*=11)*	*12407*	*1,688,819.18±1.54*
	*ST814-661 (*n*=3)*	*10385*	*1,823,975.67±2,119.17*
Farm 1	*ST6209-464 (*n*=32)*	*21553*	*1,770,576.47±616.14*
	*ST9401 (*n*=3)*	*16486*	*1,769,724.00±473.68*

### Population Structure

**Figure 2:**
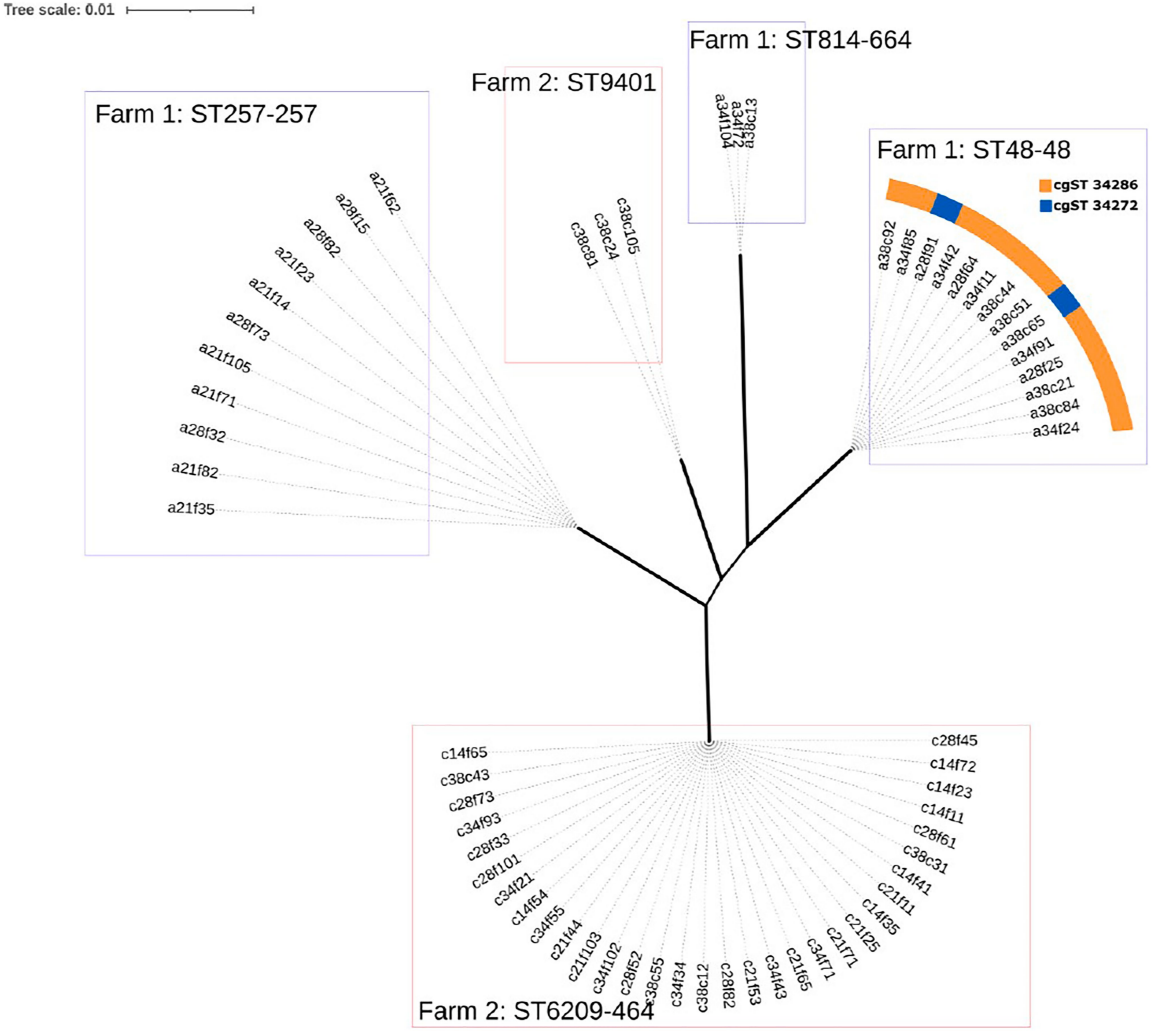
Maximum likelihood (ML) tree of the core genome alignments of isolates from Farm 1 and Farm 2 with 500 bootstraps. Branch width corresponds to bootstrap values, where wider branches correspond to higher bootstrap values that ranged from 66 to 100 between MLST types. Genomes are named to indicate farm (a=1 and c=2), broiler age (days), sample type (f=faecal and c=caecal), sample number (1 to 10) and colony number (1 to 5), i.e. ‘a21f62’.

A maximum likelihood (ML) tree was constructed using the core genome alignment of all isolates to analyse the resulting population structure (Figure 2). Clusters corresponding to each MLST type identified were detected with very short distances between genomes (Figure 2), indicating the possibility of clonal expansion. Notably, two cgST types were detected within the sequence type 48 clonal complex 48 (ST48-48) cluster, which were not evident in the ML tree (cgST-34272 and cgST-34286; Figure 2), as the corresponding alleles may not have comprised part of the core genome alignment between all strains.

### MLST/CgMLST

**Figure 3:**
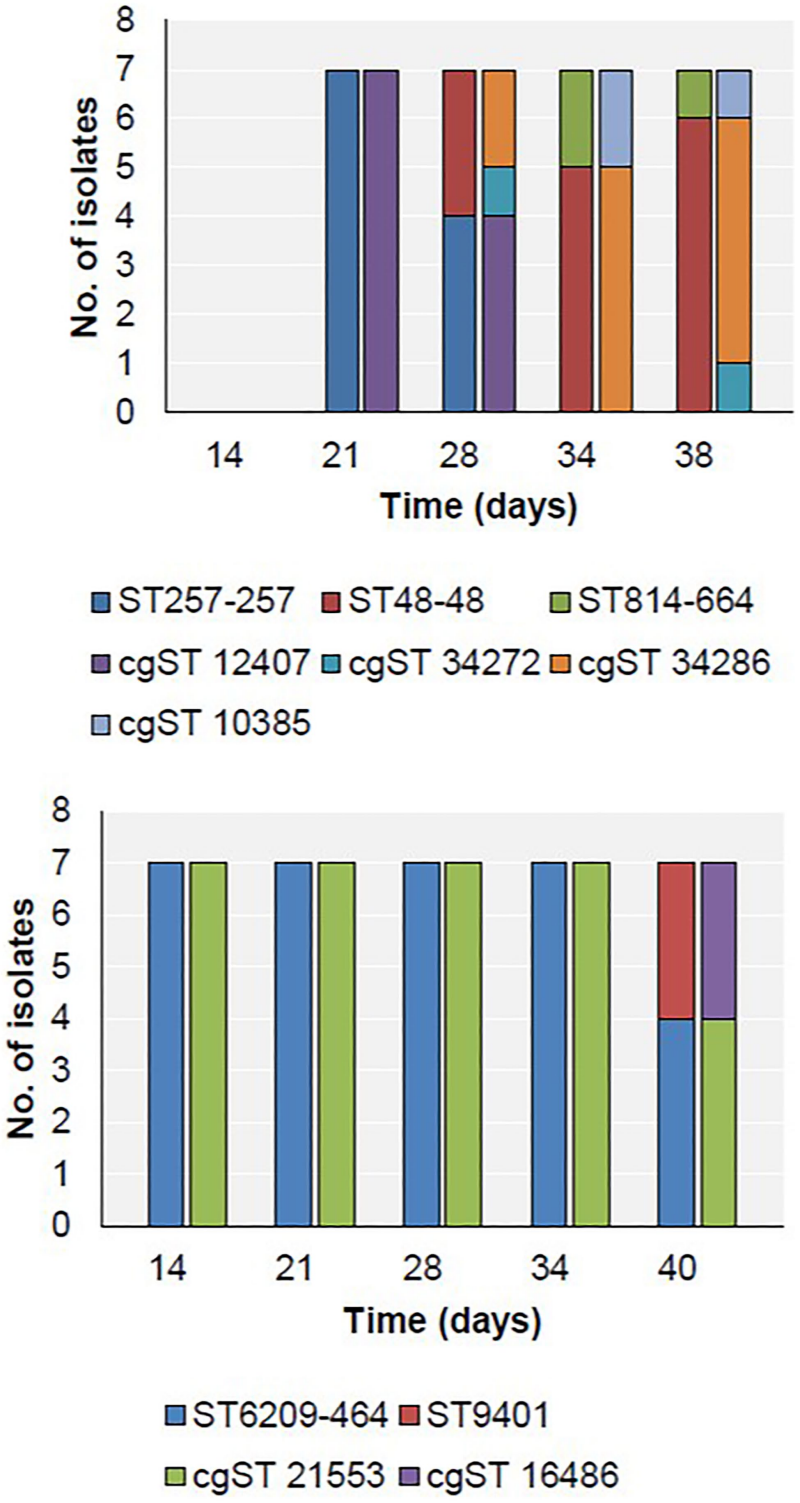
Distribution of MLST (first bar at each time point) and cgST types (second bar at each time point) in Farm 1 (top) and Farm 2 (bottom).

Three MLST types were detected in Farm 1 along with four cgST types, while two MLST types were detected on Farm 2 (Figure 3). The breakdown of alleles and profiles is provided in Appendix S1.

On Farm 1, ST257-257 (cgST-12407) was the only sequence type detected on day 21 (Figure 3). Subsequently, ST257-257 and ST48-48 were both detected on day 28. By day 34, only ST48-48 was detected, and additionally, ST814-661 was detected for the first time. These two strains persisted until slaughter on day 38. Furthermore, two cgSTs also belonging to ST48-48 were detected on days 28 and 38 – cgST-34272 and cgST-34286.

On Farm 2, the only sequence type detected during the rearing period between days 14 and 34 was ST6209-464 (cgST-21553; Figure 3). This strain persisted until slaughter on day 40, when ST9401 (cgST-16486) was also detected for the first time.

### Whole-Genome Comparison Between MLST Types

A whole-genome comparison between the MLST types detected in this study was performed and is provided in Appendix S2 along with a full list of the genes that were detected and their respective individual functional annotation.

The three MLST types detected on Farm 1 (ST257-257, ST48-48 and ST814-661) were analysed. The genes that were present in one MLST type but absent from the others were submitted for functional annotation to characterise the functional breakdown of the unique accessory genome in each of these isolates. ST814-661 was significantly associated with genes involved in cell cycle control (*p*<0.01); replication, recombination and repair (*p*<0.001); and intracellular trafficking, secretion and vesicular transport (*p*=0.001) compared to ST257-257. It was also significantly associated with a higher prevalence of genes involved in transcription (*p*<0.05); replication, recombination and repair (*p*<0.01); and intracellular trafficking, and secretion and vesicular transport (*p*<0.01) compared to ST48-48, while ST48-48 carried genes involved in inorganic ion transport and metabolism (*p*<0.05), which were not present in ST814-661.

On Farm 2, a higher prevalence of genes involved in intracellular trafficking, secretion and vesicular transport (*p*<0.05) was associated with the unique accessory genome of ST6209-464 compared to ST9401, while no significant difference was detected in the remaining COG categories.

### Virulence Gene Prevalence

**Figure 4:**
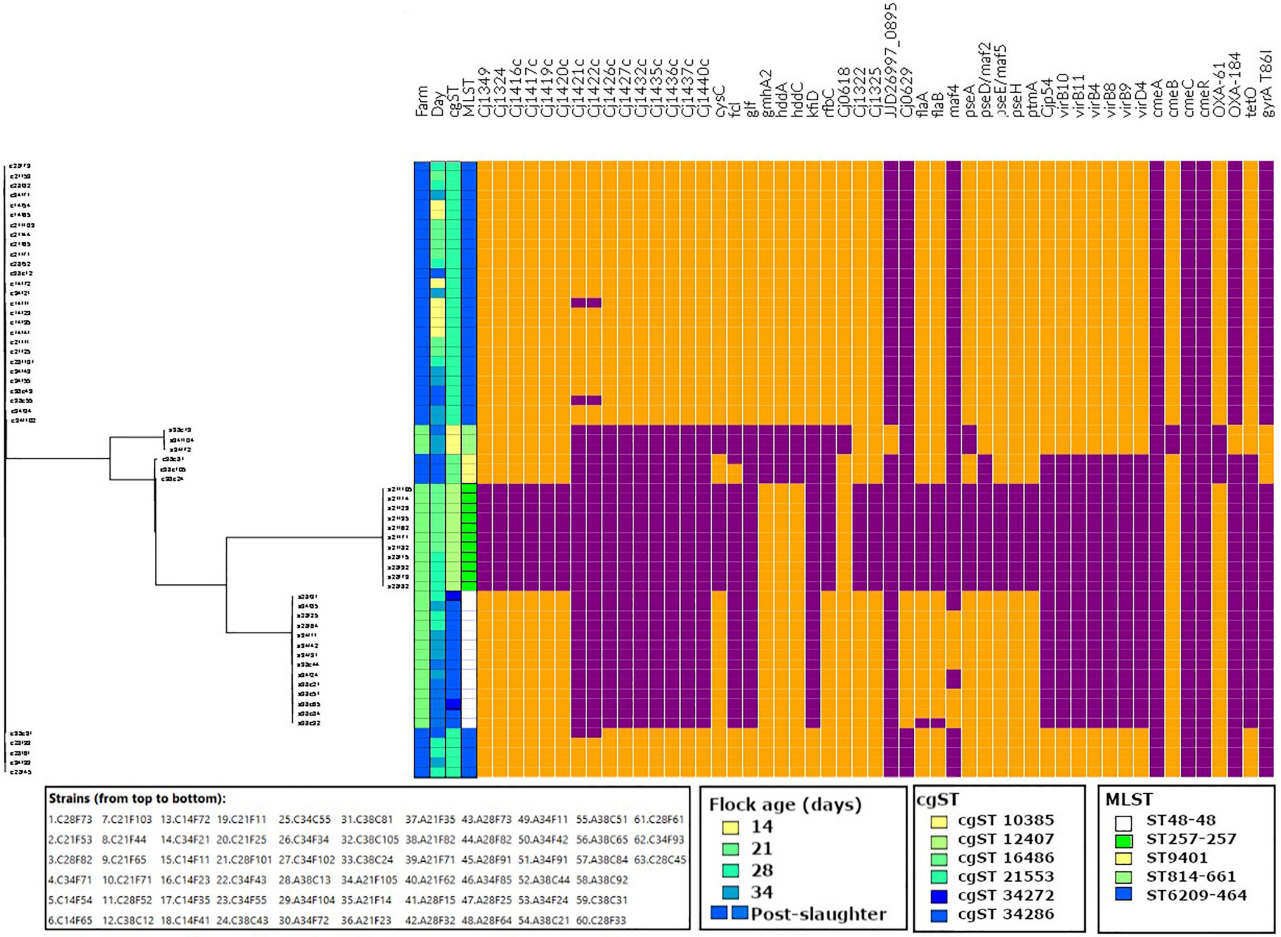
Virulence (*Cj1349* – *virD4*) and antimicrobial resistance (*cmeA* – *gyrA* T86I) profiles of the strains isolated in this study, aligned to the tree output from Roary. Data visualised on Phandango (http://jameshadfield.github.io/phandango/#/). Farm 1: green; Farm 2: blue. Present: orange; absent: purple.

In total, 153 virulence genes were detected in the 63 genomes analysed in this study (Appendix S3). Of these, 72.5% were present in all 63 genomes, which are associated with functions including adhesion, invasion, cytotoxin production, motility, chemotaxis, lipooligosaccharide synthesis, capsule synthesis, biofilm formation and colonisation. The genes that were present on Farm 1 with a prevalence ranging between 1 and 99% are shown in Figure 4.

On this farm, the prevalence of genes associated with adherence, biofilm formation, capsule, colonisation, invasion, motility and type IV secretion systems (T4SS) increased from day 21 to day 38, which corresponded with the detection of ST48-48 and ST-814-661. Interestingly, genes associated with capsule synthesis *gmhA2, hddA, hddC* and *Cj0618*, which may be associated with colonisation, were more prevalent on days 21 and 28, when ST257-257 was still detected and was less frequently found on days 34 and 38 with the recovery of ST814-661. In contrast, T4SS genes were detected on days 34 and 38 in ST814-661.

On Farm 2, no differences were observed in the prevalence of virulence genes during the broiler sampling period (days 14–34), as only one MLST type genome was detected (ST6209-464). With the exception of the absence of the capsule synthesis genes *Cj1421c* and *Cj1422c* on day 14 from one strain, the other genes were all present during the broiler production cycle. Interestingly, the prevalence of genes associated with capsule synthesis (*Cj1421c, Cj1422c, Cj1426c, Cj1427c, Cj1435c, Cj1436c, Cj1437c, Cj1440c, fcl, glf, gmhA2, hddA, hddC, kfiD* and *rfbC*), motility (*pseD/maf2*) and T4SS (*Cjp54/virB7, virB10, virB11, virB4, virB8, virB9* and *virD4*) decreased on day 38, which corresponded to the detection of ST9401. Capsule synthesis genes *Cj1421c* and *Cj1422c* were absent from one ST6209-464 isolate on day 14 and from two isolates on day 38. Additionally, two out of three ST9401 isolates carried the capsule synthesis gene *fcl*. Overall, no change in the prevalence of virulence genes was observed during the broiler rearing period in Farm 2; however, a decrease in these virulence genes was observed with the detection of ST9401 at slaughter.

### Antimicrobial Resistance

Antimicrobial resistance was measured phenotypically, and the prevalence of genomic antimicrobial resistance determinants was characterised for each isolate (Figure 4).

Antimicrobial resistance was not detected in isolates from Farm 1 until day 34, with the isolation of ST814-661. On day 34, resistance to TET was detected in one isolate, while on day 38, one ST814-661 isolate was phenotypically resistant to both TET and CIP.

In contrast, on Farm 2, all isolates detected during the broiler rearing period exhibited resistance to TET (ST6209-464), while on day 40, a decrease in the prevalence of TET resistance was observed, with the detection of TET ST9401, which was susceptible to TET. Furthermore, two ST9401 isolates recovered on day 40 exhibited phenotypic resistance to CIP. All strains except ST814-661 carried *cmeABC* and *cmeR*, which encodes the multidrug efflux system (Figure 4).

There was complete agreement between the prevalence of the TET resistance gene *tetO* and TET resistance in ST6209, while a higher recovery rate of *tetO* was detected in ST814-661 compared to that observed in phenotypic susceptibility tests. Similarly, the fluoroquinolone resistance amino acid substitution *gyrA* T86I was more prevalent than phenotypic resistance to CIP, as it was found in all ST814-661 and ST9401 isolates, as compared to one out of three ST814-661 and two out of three ST9401 that exhibited resistance phenotypically. Additionally, isolates that carried *tetO* but did not exhibit phenotypic resistance showed reduced susceptibility as compared to the isolates that were *tetO* negative.

ST814-661 carried *cmeA*, *cmeC* and *cmeR*, while *cmeB* was detected with less than 80% identity and thus filtered out of the analysis. Additionally, all strains except ST9401 carried *bla_OXA-61_*, which is associated with β-lactam resistance. *bla_OXA-61_* was the most frequently detected *bla_OXA_* gene, present in ST257-257, ST48-48 and ST6209, while *bla_OXA-184_* was found in ST814-661.

## Discussion

In this study, we analysed the progression of *C. jejuni* sequence types during rearing on two commercial broiler farms in Ireland. The genomic proximity of same-MLST type strains indicates the possibility of clonal expansion in the broiler house (Figure 1). In Farm 1, three different MLST types were identified. ST48-48 and ST814-661 persisted until slaughter, while ST257-257 was only detected on days 21 and 28. This indicates the possibility that ST257-257 may have been outcompeted by the other two strains or that it was below the level of detection of the sampling strategy applied in this study. In the PubMLST database, ST257-257 and ST48-48 are most commonly recovered from humans, chicken and cattle, while ST814-661 is most commonly recovered from humans and chickens (Jolley et al., 2018), although this could reflect a submission bias. Additionally, the presence of two cgST types within ST48-48 in this study (cgST-34286 and cgST-34272) early in the production cycle indicates that two distinct types belonging to ST48-48 may have colonised this flock. Clonal complex ST-48 was previously found in a wide range of patients, including reactive arthritis patients, and often in association with fluoroquinolone resistance (Nielsen et al., 2010; Collado et al., 2018). In contrast, in 2012, it was associated with fluoroquinolone susceptibility in the United Kingdom, which was also observed in this study (Cody et al., 2012). Furthermore, clonal complex ST-257 was previously reported to be associated with hospitalisation of infected humans (Harvala et al., 2016).

In Farm 2, ST6209-464 was found during the rearing cycle. There are only twenty-seven records of previously recovered ST6209-464 in PubMLST, all of which were from humans or chickens (Jolley et al., 2018). All ST6209-464 isolates in this study were TET resistant, which reflects a recent study that reported TET and CIP resistance is common among ST6209-464 isolates in Lithuania (Aksomaitiene et al., 2019). During slaughter, ST9401 was also detected and carried the fluoroquinolone resistance determinant *gyrA* T86I. This MLST type has rarely been reported, twice associated with humans and once in chicken samples (Jolley et al., 2018).

Overall, two distinct patterns of *Campylobacter* detection were observed on Farms 1 and 2, whereby multiple MLST types were identified during the production cycle in the former and only one was found in the latter. The replacement of a dominant strain during broiler production has been previously reported, reflecting the dynamics observed on Farm 1 (Bull et al., 2006). In contrast, it has also been reported that a predominant strain can persist throughout the production cycle and after slaughter, reflecting the results observed on Farm 2 (Inglis et al., 2020). The difference in the diversity of MLST strains across the two farms could reflect individual farm practices including differences in biosecurity compliance, adjacent local environmental reservoirs including livestock and wildlife and/or antimicrobial interventions or may be a seasonal factor (Jorgensen et al., 2011; Battersby et al., 2016; Meunier et al., 2016). Indeed, Farm 2 was sampled while COVID-19 and avian influenza restrictions were operational, which may have contributed to increased biosecurity in this farm as compared to Farm 1, resulting in less opportunity for different *Campylobacter*s to access the flock, as previously observed (DAFM, 2020; HPSC, 2020). Notably, the detection of ST814-661 in Farm 1 and ST9401 in Farm 2 samples corresponds with thinning and slaughter, respectively, which suggests that these practices may still pose a risk of contamination, as previously reported (Koolman et al., 2014; Prachantasena et al., 2016).

We detected 153 virulence genes in total in this study (Appendix S3). Most of these genes were detected in all isolates that were tested, which includes the ubiquitous presence of virulence genes associated with adhesion and invasion (*ciaB, cadF and pldA*) and cytotoxin production (*cdtABC*). Thus, all of the isolates identified in this study have the potential to cause human illness (Konkel et al., 1999; Ziprin et al., 2001; Flanagan et al., 2009; Lai et al., 2016; Scuron et al., 2016). This is supported by evidence that isolates belonging to these MLST types have been previously isolated from humans (Jolley et al., 2018). On Farm 1, genes associated with colonisation, capsule synthesis, motility, biofilm formation, adhesion and T4SS were prevalent in isolates that persisted until the end of the broiler production cycle (ST48-48 and ST814-661). Bacterial T4SS contributes to the delivery of virulence factors into eukaryotic or prokaryotic cells. In *Campylobacter* spp., T4SS can contribute to the activity of adhesins and invasins, such as *Campylobacter* invasion antigen (Cia) proteins (Bacon et al., 2000; Sgro et al., 2019). ST6209-464, which was the only strain detected on Farm 2 during the production cycle, carried the highest number of virulence genes of any strain in this study, carrying between 148 and 150 virulence determinants, while the remaining isolates tested positive for between 114 and 133 virulence determinants. Overall, the strains with the greatest number of virulence determinants in this study persisted until the end of the production cycle (ST48-48, ST814-661 and ST6209-464).

On Farms 1 and 2, more antimicrobial resistance determinants were detected towards the end of the production cycle and at slaughter, which in Farm 1 corresponded with the detection of *tetO* and *gyrA* T86I-carrying ST814-661, and in Farm 2 corresponded with the detection of *gyrA* T86I-positive ST9401. Furthermore, the prevalence of phenotypic TET resistance in ST6209-464 was consistent with the detection of *tetO*, and a high level of concordance between phenotypic and genotypic resistance was also observed for *tetO* in ST814-661 for two of the three strains that were TET resistant. The third isolate exhibited reduced susceptibility, while *gyrA* T86I was detected in six strains, three belonging to ST9401 and three from ST814-661, with 50% concordance with the observed phenotypic results. Notably, ST814-661 strains did not carry the complete *cmeB* gene that encodes a multidrug efflux pump, which may have contributed to reduced TET and fluoroquinolone resistance, despite the presence of *tetO* and *gyrA* T86I (Pumbwe and Piddock, 2002). Comparable levels of *gyrA* T86I carriage and CIP resistance concordance have also been previously reported (Wysok et al., 2020).

While significant differences in the prevalence of genes associated with specific biological functions were identified between MLST types (Appendix S2), no COG categories were over-represented in ST48-48 as compared to ST257-257. In contrast, inorganic ion metabolism and transport were more prevalent in ST48-48 compared to ST814-661. It was previously suggested that this functional category is under selective pressure in *Vibrio* spp. using gene ontology enrichment analysis; however, further investigation is needed to confirm whether this category is important for the persistence of *Campylobacter* spp. in broilers (Rabby et al., 2015). The same study also proposes that COG categories, such as intracellular trafficking and secretion, may be under selective pressure due to their role in host-pathogen interactions and nutrition acquisition and metabolism. It has also been previously suggested that variable distributions of accessory genes between strains can be associated with ecological niche adaptation (McInerney et al., 2017), which has been observed in accessory genes from *Staphylococcus haemolyticus* that exhibited variable distribution across a variety of niches, including human, copper, willow and rice niches (Chaudhry and Patil, 2020). Similarly, *C. jejuni* carries accessory genes that are differentially distributed across strains, indicating that a similar form of ecological adaptation may occur (Park et al., 2020).

## Conclusion

In this study, we investigated changes in *C. jejuni* populations during broiler rearing on two Irish broiler farms. We identified five MLST types and six cgSTs across the two farms. The first detection of two of the MLST types (ST814-661 and ST9401) corresponded with the time of thinning and slaughter, respectively. More antimicrobial resistance and virulence determinants were detected in isolates at slaughter than during the early stages of the production cycle. Pangenomic analysis revealed that the most frequently detected strains (ST48-48 and ST6209-464) carried more genes associated with COG categories including intracellular trafficking and inorganic ion metabolism and transport compared to less frequently detected strains (ST814-661 and ST9401, respectively). Overall, our results indicate a possible relationship between broiler rearing and changes in *C. jejuni* populations that could be of public health significance; however, further investigation with larger sample sizes, more farms and under different conditions is required to firmly establish if this observation holds true in the broiler production environment.

## Data Availability Statement

The datasets generated for this study can be found under BioProject ID PRJNA688841.

## Author Contributions

DB obtained the funding. All authors were responsible for conceiving and the design of the study. BT acquired, analysed, and interpreted the data. BT drafted the manuscript while PW, CB, and DB did the critical revision.

## Funding

This research was funded by the Teagasc (the Agriculture and Food Development Authority for Ireland) under the Teagasc Walsh Scholarship scheme (project number 0028).

## Conflict of Interest

The authors declare that the research was conducted in the absence of any commercial or financial relationships that could be construed as a potential conflict of interest.

## Publisher’s Note

All claims expressed in this article are solely those of the authors and do not necessarily represent those of their affiliated organizations, or those of the publisher, the editors and the reviewers. Any product that may be evaluated in this article, or claim that may be made by its manufacturer, is not guaranteed or endorsed by the publisher.

## Footnotes

^1^ https://github.com/tseemann/mlst

^2^ https://github.com/aldertzomer/cgmlst

^3^ https://github.com/tseemann/abricate

## Supplementary Material

APPENDIX S1 MLST and cgMLST allelic profiles.

APPENDIX S2 Functional annotation of differences between the gene content of MLST strains.

APPENDIX S3 Detection of virulence and antimicrobial resistance determinants.
