# Supplementary material for: Genomic Characterisation of Campylobacter jejuni Isolates Recovered During Commercial Broiler Production
Source: Front Microbiol. 2021 Oct 14;12:716182. doi: 10.3389/fmicb.2021.716182 (PMC8552067; doi:10.3389/fmicb.2021.716182)
Supplement: APPENDIX S1 — MLST and cgMLST allelic profiles. [file Data_Sheet_1.ZIP › Supplementary materials.zip!/S2 Functional annotation/S2.2 Whole genome comparison/Appendix S2.2.docx]

**Appendix S2.2:** Whole genome comparison of the unique accessory genome between the MLST types from each farm, where each column represents genes from the first MLST type that were absent from the second MLST type which were grouped by COGs. The COG categories shown are: B (chromatin structure), C (energy production and conversion), D (cell cycle control), E (amino acid transport and metabolism), F (nucleotide transport and metabolism), G (carbohydrate transport and metabolism), H (coenzyme transport and metabolism), I (lipid transport and metabolism), J (translation, ribosomal structure and biogenesis), K (transcription), L (replication, recombination and repair), M (cell wall/membrane biosynthesis), N (motility), O (post-translational modification, protein turnover and chaperones), P (inorganic ion transport and metabolism), Q (secondary metabolite biosynthesis, transport and catabolism), S (function unknown), T (signal transduction), U (intracellular trafficking, secretion and vesicular transport), V (defence mechanisms) and “Uncharacterised”.

| COG Category | ST257 vs ST48 (n = 158) | ST48 vs ST257 (n = 141) | ST257 vs ST814 (n = 204) | ST814 vs ST257 (n = 309) | ST48 vs ST814 (n = 177) | ST814 vs ST48 (n = 299) | ST6209 vs ST9401 (n = 182) | ST9401 vs ST6209 (n = 173) |
| --- | --- | --- | --- | --- | --- | --- | --- | --- |
| B | 0 (0.00%) | 1 (0.71%) | 0 (0.00%) | 0 (0.00%) | 1 (0.56%) | 0 (0.00%) | 0 (0.00%) | 0 (0.00%) |
| C | 6 (3.80%) | 8 (5.67%) | 4 (1.96%) | 6 (1.94%) | 9 (5.08%) | 9 (3.01%) | 7 (3.85%) | 6 (3.47%) |
| D | 0 (0.00%) | 2 (1.42%) | 0 (0.00%) | 7 (2.27%) | 1 (0.56%) | 6 (2.01%) | 1 (0.55%) | 1 (0.58%) |
| E | 6 (3.80%) | 12 (8.51%) | 10 (4.90%) | 10 (3.24%) | 9 (5.08%) | 3 (1.00%) | 7 (3.85%) | 9 (5.20%) |
| F | 0 (0.00%) | 2 (1.42%) | 1 (0.49%) | 1 (0.32%) | 3 (1.69%) | 1 (0.33%) | 2 (1.10%) | 2 (1.16%) |
| G | 9 (5.70%) | 5 (3.55%) | 15 (7.35%) | 12 (3.88%) | 11 (6.21%) | 12 (4.01%) | 12 (6.59%) | 12 (6.94%) |
| H | 4 (2.53%) | 2 (1.42%) | 8 (3.92%) | 3 (0.97%) | 5 (2.82%) | 2 (0.67%) | 3 (1.65%) | 4 (2.31%) |
| I | 6 (3.80%) | 3 (2.13%) | 4 (1.96%) | 5 (1.62%) | 2 (1.13%) | 4 (1.34%) | 6 (3.30%) | 7 (4.05%) |
| J | 5 (3.16%) | 3 (2.13%) | 7 (3.43%) | 6 (1.94%) | 4 (2.26%) | 5 (1.67%) | 5 (2.75%) | 3 (1.73%) |
| K | 3 (1.90%) | 1 (0.71%) | 6 (2.94%) | 13 (4.21%) | 5 (2.82%) | 14 (4.68%) | 4 (2.20%) | 10 (5.78%) |
| L | 6 (3.80%) | 6 (4.26%) | 3 (1.47%) | 20 (6.47%) | 9 (5.08%) | 26 (8.70%) | 18 (9.89%) | 10 (5.78%) |
| M | 20 (12.66%) | 28 (19.86%) | 22 (10.78%) | 26 (8.41%) | 24 (13.56%) | 20 (6.69%) | 15 (8.24%) | 15 (8.67%) |
| N | 5 (3.16%) | 4 (2.84%) | 5 (2.45%) | 5 (1.62%) | 2 (1.13%) | 4 (1.34%) | 8 (4.40%) | 3 (1.73%) |
| O | 1 (0.63%) | 2 (1.42%) | 2 (0.98%) | 2 (0.65%) | 2 (1.13%) | 1 (0.33%) | 2 (1.10%) | 4 (2.31%) |
| P | 2 (1.27%) | 7 (4.96%) | 17 (8.33%) | 9 (2.91%) | 21 (11.86%) | 8 (2.68%) | 16 (8.79%) | 12 (6.94%) |
| Q | 10 (6.33%) | 9 (6.38%) | 12 (5.88%) | 11 (3.56%) | 6 (3.39%) | 4 (1.34%) | 5 (2.75%) | 4 (2.31%) |
| S | 40 (25.32%) | 23 (16.31%) | 49 (24.02%) | 89 (28.80%) | 34 (19.21%) | 91 (30.43%) | 36 (19.78%) | 48 (27.75%) |
| T | 4 (2.53%) | 3 (2.13%) | 2 (0.98%) | 1 (0.32%) | 3 (1.69%) | 3 (1.00%) | 6 (3.30%) | 1 (0.58%) |
| U | 4 (2.53%) | 7 (4.96%) | 10 (4.90%) | 31 (10.03%) | 13 (7.34%) | 31 (10.37%) | 15 (8.24%) | 6 (3.47%) |
| V | 4 (2.53%) | 8 (5.67%) | 8 (3.92%) | 7 (2.27%) | 10 (5.65%) | 5 (1.67%) | 6 (3.30%) | 6 (3.47%) |
| Uncharacterised | 29 (18.35%) | 10 (7.09%) | 30 (14.71%) | 59 (19.09%) | 15 (8.47%) | 63 (21.07%) | 23 (12.64%) | 22 (12.72%) |
